# Shall the Entrance Standard Be Raised?

**Published:** 1894-11

**Authors:** 


### Editorial.

### SHALL THE ENTRANCE STANDARD BE RAISED?

The effort to raise the standard of entrance to medical and dental colleges has been the topic of discussion in interested circles for years. The solution of this problem for the United States seems no nearer to-day than at any time within the past two decades. While this is true, the pressure in certain directions for a higher education has become so persistent and powerful that it seems necessary that the question should be discussed more thoroughly than heretofore.

There can be no antagonistic views held in regard to the general proposition that in proportion to an advanced educational training will be the capacity of the individual for further acquirement, but there must be room for disagreement in regard to the measures necessary to reach this desirable condition.

An advance by leaps and bounds is not healthy progress in any line of work or mental growth, and those who become impatient of results and would have the problem solved in resolutions in societies are not wise in their generation, and, if not checked, become stumbling-blocks in the way of real advancement.

At the last meeting of the American Dental Association, and also at that of the National Association of Dental Faculties, the question of a higher standard formed an important topic. From the proceedings of the former are quoted, on another page, the main points of the report of the Section on Education, together with the discussion which followed. The general remarks on the same subject in the Association of Faculties are not reported, but the resolutions introduced last year and laid over for action this, requiring for entrance to the first year physics and Latin, were warmly discussed and at first passed by a bare majority, to be laid on the table at the next session, on a vote of reconsideration.

The feeling manifested at both of these meetings indicates that a large body, perhaps a majority, of the educators of the United States are dissatisfied with the present condition of things, and are anxious for a change. This is encouraging if it be regarded in the light of aspiration after something better, but the possibility of attainment must always be the factor to be considered in any move, whether it be in the individual or in the community.

The Section on Education reported to the American Dental Association that it "would urge that the degree of Bachelor of Arts, or its equivalent, obtained from a reputable university, should be required."

If it were possible to enforce this, the result would doubtless be the practical destruction of every medical and dental school in this part of North America. In this country institutions of learning, classical, scientific, and professional, are not, as a rule, under the control of the government, State or national. Some few of the States have established universities and can regulate the standard, but this is so exceptional as not to be regarded as a factor in the calculation. It would no doubt be an excellent thing to have all educational institutions under the management of the central government at Washington, as is done in Europe; but are the people of the United States prepared to adopt this? Could it be carried out under the crude conditions of life existing in many sections? The answer, it seems, must be positively in the negative.

Unfortunately for the hopes of those who look forward to the day when the degree of Bachelor of Arts shall be required for entrance to the professions, fewer men holding this degree are entering those of medicine and dentistry to-day than heretofore.

Dr. J. M. Da Costa pointed out in his address before the Howard Medical Alumni Association that the percentage of college graduates in Howard fell from 53.9 in 1884 to 28.2 in 1892, and has been since declining. At the University of Michigan in ten years, from 1882 to 1892, the proportion in the Medical School at Ann Arbor fell from 10.6 to 9.4 per cent. At the University of Pennsylvania the proportion was twenty-six in 1890–91, and fell in three years to twenty per cent. According to the authority quoted, "sixteen medical colleges were last year without a single college-graduate on their rolls."

While this is doubtless true, and to many is a discouraging fact, it does not mean, as it seems to imply, that the large majority, who had not college educations, were illiterate and incapable of professional instruction. The larger proportion of these had higher school training, and when not thus far advanced, it has covered the solid foundation of that of the public schools, which, while not including the languages, furnishes an excellent basis for practical work and theoretical knowledge. It is not to be understood that this statement of fact means an opinion that nothing further is desirable; on the contrary, the writer is anxious for the period to arrive when it will be possible to secure a higher culture; but this condition, in his opinion, has not yet been attained.

When the public schools will begin a training in the languages, including Latin at the ninth year, and continue this, without interruption, to the seventeenth year, the professional schools can advance the present standard with safety to themselves and with justice to the students, and not until then.

Professor Taft, in his remarks on the occasion referred to, states that "ten years ago more than one-half the practitioners in dentistry were very defective in their ability to use their native language." We would hesitate to credit the accuracy of this statement from any other man, but coming from one of his long and wide observation as an educator and journalist, it must be accepted as true in his experience. It, however, does not accord with the writer's observation of the portion of the dental profession he is familiar with. If, as he further stated, this is "not true to-day," it demonstrates a very satisfactory improvement in the methods of training the rising generation.

It is not possible to reach the statistics of all the dental schools of the United States, but it is interesting to note that in one of the large dental schools of Philadelphia the records show for the past four sessions that sixty-three per cent. entered from the public schools and upon examinations, and that thirty-seven per cent. came from high schools and colleges. Whether this will hold good for all dental colleges in the United States we have no means of determining, but it is assumed the average per cent. will be nearly the same.

Taking this for granted, it implies that if the sixty-three per cent. were deprived of the privilege of entering these schools, it would leave so small a proportion of students that only those institutions supported by government endowments could be sustained. If, then, the law as proposed in the National Association of Dental Faculties be carried out next year, it will either end in the destruction of two-thirds of the colleges or will result in what is far worse, a persistent neglect on the part of schools to carry out its requirements. While some may regard the crippling if not the total destruction of the means of professional education as not a serious evil, we must so regard it in view of the necessities of the extended territory and increasing population of the country.

It must be clear, therefore, that European methods of preliminary training are not yet adapted to the comparatively new conditions here existing, and it must be conceded that the necessity for skilled practitioners overrides that for men of extended culture.

While it may be true in a general way that "differences in manipulative ability do not depend on degrees of general education," it is certainly true that in proportion to the development in a higher classical training is there an equal loss of manipulative ability. This must have been clearly manifested to all dental educators,—certainly so to the writer.

It seems, therefore, that we must wait the slow development of time before we can hope to have a standard equal to our desires, or one that will draw a proper line between the inferior and superior development of the intellectual faculties. That this period will surely come as a professional standard there can be no doubt, but it can only be by the slow and healthy development common in all advanced civilizations.

